# Role of microRNAs in Disorders of Gut–Brain Interactions: Clinical Insights and Therapeutic Alternatives

**DOI:** 10.3390/jpm11101021

**Published:** 2021-10-12

**Authors:** Rajan Singh, Hannah Zogg, Seungil Ro

**Affiliations:** Department of Physiology and Cell Biology, Reno School of Medicine, University of Nevada, 1664 North Virginia Street, Reno, NV 89557, USA; rajans@med.unr.edu (R.S.); hannahzogg@gmail.com (H.Z.)

**Keywords:** functional dyspepsia, irritable bowel syndrome, gastroparesis, slow transit constipation, neuroimmune interaction, gut barrier function, visceral hypersensitivity, serotonin, miRNA therapeutics

## Abstract

Disorders of gut–brain interactions (DGBIs) are heterogeneous in nature and intertwine with diverse pathophysiological mechanisms. Regular functioning of the gut requires complex coordinated interplay between a variety of gastrointestinal (GI) cell types and their functions are regulated by multiple mechanisms at the transcriptional, post-transcriptional, translational, and post-translational levels. MicroRNAs (miRNAs) are small non-coding RNA molecules that post-transcriptionally regulate gene expression by binding to specific mRNA targets to repress their translation and/or promote the target mRNA degradation. Dysregulation of miRNAs might impair gut physiological functions leading to DGBIs and gut motility disorders. Studies have shown miRNAs regulate gut functions such as visceral sensation, gut immune response, GI barrier function, enteric neuronal development, and GI motility. These biological processes are highly relevant to the gut where neuroimmune interactions are key contributors in controlling gut homeostasis and functional defects lead to DGBIs. Although extensive research has explored the pathophysiology of DGBIs, further research is warranted to bolster the molecular mechanisms behind these disorders. The therapeutic targeting of miRNAs represents an attractive approach for the treatment of DGBIs because they offer new insights into disease mechanisms and have great potential to be used in the clinic as diagnostic markers and therapeutic targets. Here, we review recent advances regarding the regulation of miRNAs in GI pacemaking cells, immune cells, and enteric neurons modulating pathophysiological mechanisms of DGBIs. This review aims to assess the impacts of miRNAs on the pathophysiological mechanisms of DGBIs, including GI dysmotility, impaired intestinal barrier function, gut immune dysfunction, and visceral hypersensitivity. We also summarize the therapeutic alternatives for gut microbial dysbiosis in DGBIs, highlighting the clinical insights and areas for further exploration. We further discuss the challenges in miRNA therapeutics and promising emerging approaches.

## 1. Introduction

Disorders of gut-brain interactions (DGBIs), including functional dyspepsia (FD) and irritable bowel syndrome (IBS), are common conditions both in the community and in clinical practice [1,2]. The worldwide prevalence of DGBIs is approximately 40% based on a recent study using Rome IV diagnostic criteria [1]. The clinical spectrum of DGBI symptoms includes nausea, fullness, bloating, abdominal pain/burning, vomiting, and changes in bowel habits [3,4]. Furthermore, DGBIs and specific gut motility disorders often present significant clinical overlaps (i.e., gastroparesis and FD-postprandial distress syndrome (PDS) (90%), gastroparesis and slow transit constipation (STC) (60%), and FD and IBS (15–42%)) [5,6,7]. The current treatment options are lifestyle modification, dietary intervention, probiotics, laxatives, antispasmodics, prokinetics, and centrally acting neuromodulators [2,8,9]. However, relieving symptoms is not enough, therefore there is an immense demand to elucidate effective treatment regimens for DGBIs based on the underlying pathophysiology. The understanding of the pathogenesis of DGBIs and gut motility disorders has evolved as the pathophysiological mechanisms have been exposed at the cellular and molecular level [10,11,12,13]. Not long ago, “functional” gastrointestinal (GI) disorders, recently classified as DGBIs, were explained as idiopathic, and the patients were believed to be neurotic, apprehensive, and otherwise healthy individuals with an imaginary illness by both physicians and researchers [14]. By now, with enhanced pathophysiological knowledge, DGBIs are being increasingly understood to be complex, heterogeneous, and multifactorial disorders [2,9,15,16]. The symptom-based criteria for DGBIs in the absence of alarming features (i.e., unexplained weight loss and severe abdominal pain) and observation of normal looking GI mucosa during endoscopy do not rule out abnormalities at the cellular level, which is critical in underpinning the pathogenesis.

Normal gut functions require a complex coordinated interplay between the gut and brain, which necessitates a variety of cell types in the GI tract, for instance, interstitial cells of Cajal (ICCs), enteric neurons, smooth muscle cells (SMCs), enteroendocrine cells, enterocytes, and immune cells [17,18]. Further, functional defects of these cells lead to alternations in gut physiology and pathophysiology [10,19,20,21,22,23]. Normal functioning of cells in the GI is regulated by molecular mechanisms at the transcriptional, post-transcriptional, translational, and post-translational levels [23]. MicroRNAs (miRNAs) are small non-coding RNAs that regulate gene expression post-transcriptionally and modulate pathophysiological mechanisms of DGBIs [24]. miRNAs have been linked to gut physiological mechanisms such as visceral sensation (miR-200a, -199a/b, -338, and -495), gut immune functions (miR-29, -155, -146a/b, -192, -146a, -155, and -122), GI barrier functions (miR-16, -29a, -219a, and -122a), neuronal cell development and function (miR-375), as well as gut motility (miR-10b, -143, -551b, -222, -145, let-7f, -375, and -128) [23,25,26,27,28,29,30,31]. These mechanisms are highly relevant to the gut where neuroimmune interactions are key contributors to the control of gut functions and interruptions in these interactions lead to DGBIs [32,33,34,35]. Thus, therapeutic modalities utilizing naturally occurring miRNAs represent a very promising approach for the clinical care of DGBIs. The use of miRNA-based therapeutics has dual advantages [36,37,38]. First, miRNAs are naturally occurring molecules in human cells, unlike man-made chemotherapy compounds and therefore have all the mechanisms in place for their processing and downstream target selection. Second, miRNAs act by targeting multiple genes within one pathway, thus causing a broader yet specific response. miRNA-based therapeutics could therefore represent a promising alternative to existing RNA-based therapies and may potentially boost therapeutic effects compared with synthetic siRNAs that influence only a single target gene; however, the translation of miRNA-based therapeutics into the clinic has been hampered by issues associated with off-target effects and inefficient delivery mechanisms [38]. In line with this, clinical trials have been terminated most often due to these issues. Thus, miRNAs will only be suitable for therapeutic development if toxicities are carefully assessed and delivery methods are improved [37,38]. In this review, we discuss the impact of miRNAs in pathogenesis of DGBIs, the key challenges facing miRNA therapeutics, and promising emerging approaches.

## 2. Functional Role of miRNAs in Gut Physiological Mechanisms

**Figure 1:**
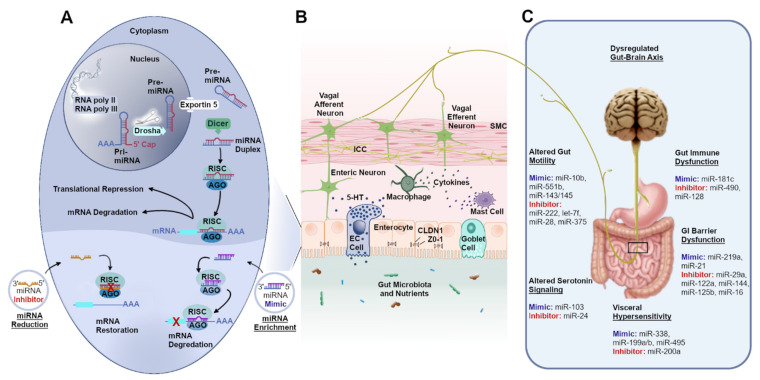
miRNA mediated pathophysiological mechanisms and potential therapeutic alternatives for DGBIs. (**A**). miRNA biogenesis and miRNA therapeutic approaches to reduce or enhance miRNA levels leading to mRNA restoration or mRNA degradation/translational repression, respectively, (**B**). Dysregulated miRNAs are evidenced in gastrointestinal pacemaking cells, immune cells, intestinal epithelial cells, and enteric neurons, (**C**). Possible miRNA therapeutics based on pathophysiological mechanisms. Abbreviations: AGO, argonaute; CLDN1, claudin-1; EC cell, enterochromaffin cell; ICC, interstitial cell of Cajal; miRNA, microRNA, mRNA, messenger RNA; RISC, RNA-induced silencing complex; SMC, smooth muscle cell; ZO-1, zonula occludens; 5-HT, 5-hydroxytryptamine.

miRNAs are small non-coding RNAs that act as post-transcriptional regulators of cellular processes [39] and function to regulate protein expression in all cells [40], including those in the GI [30,41]. Therefore, miRNAs are critical for modulating gut physiological mechanisms. miRNA biogenesis is a multistep process involving, the production of long primary miRNA transcripts (pri-miRNA) by RNA polymerases II and III (Figure 1A) [42,43]. The pri-miRNA molecule forms a hairpin structure, which is recognized by Drosha, a microprocessor complex containing the nuclear RNase III endonuclease that processes the pri-miRNA into a nucleotide stem loop RNA [42,44]. This precursor miRNA (pre-miRNA) is transported from the nucleus, via exportin 5 into the cytoplasm [45]. The pre-miRNA is further processed by Dicer, a RNAse III endonuclease, which cleaves the loop and results in the formation of a double-stranded miRNA duplex containing a 5′ miRNA and a 3′ miRNA [43,46]. The miRNA duplex associates with the RNA-induced silencing complex (RISC), where a single-stranded mature miRNA is selected [38,43,44]. The seed region of the mature miRNA complements to the 3′untranslated region of the target messenger RNA (mRNA) sequence based on base-pair stability [47]. Further, miRNA associated with an Argonaute (AGO) protein forms RISC and AGO facilitates destabilization and/or translational repression of the associated transcripts (Figure 1A) [38,42,43]. The functional role of miRNAs is a complex and tightly regulated task to modulate targets at the post-transcriptional (translational) level [42,48,49]. Previous studies evidenced that loss of functional activity of the GI pacemaking cells including, ICCs, SMCs, enteric neurons, platelet-derived growth factor receptor alpha positive (PDGFRα^+^), and immune cells are driving forces contributing to the pathogenesis of DGBIs [10,23,29,31,50]. Cellular dysfunction in GI cells mediated via dysregulation of miRNAs likely regulates a variety of gut physiological and pathophysiological mechanisms leading to the development of diseases such as DGBIs [51,52].

Approximately 60% of protein coding mRNAs are modulated by miRNAs, including many of the key proteins associated with diseases [41,53]. In recent studies, the impacts of miRNAs have been exhibited to regulate pathophysiological mechanisms of DGBIs, including impaired GI barrier function, gut immune dysfunction, visceral hypersensitivity, and gut dysmotility [26,28,29,31,51]. The identification of precise miRNA targets has been the topic of extensive research. Additionally, there is emerging evidence that the full impact of some miRNAs upon their targets may only be seen in very specific disease conditions or in response to certain environmental stimuli [54]. By elucidating miRNA-mediated pathogenesis in DGBIs, researchers hope to elucidate target molecules that can prevent and/or reverse key steps in disease development. miRNAs are of potential importance for new therapeutic approaches due to their ability to target an entire pathway rather than a single gene, making the clinical outcomes much more substantial than other therapeutic molecules. Thus, exploring the roles of miRNAs in cellular apoptotic and differentiation pathways in GI pacemaking cells and a better understanding of the role of miRNAs in gut pathological mechanisms might unearth novel therapeutic clues of which miRNA mimics/inhibitors may restore the functional activity of damaged cells causing DGBIs.

## 3. Impact of miRNAs in DGBIs

DGBIs are heterogeneous in nature with diverse pathophysiological mechanisms. miRNAs mediate cellular and molecular remodeling in GI pacemaking cells, immune cells, and enteric neurons (Figure 1B) (Table 1). Additionally, they may be responsible for pathophysiological mechanisms of DGBIs (Figure 1C) (Table 1).

### 3.1. miRNA Dysregulation in GI Cells Contribute to the Pathogenesis of DGBIs

Delayed gut transit is result of irregular GI muscular movements, which is a major pathophysiological mechanism of DGBIs [10,23]. Recent studies have elucidated the cellular and molecular alterations underpinning the abnormalities in GI cells, which are crucial for gut homeostasis [23,28,29]. Further exploration of the precise miRNA-mediated pathologies contributing to the cellular plasticity in DGBIs is needed. We have reviewed recent literature on miRNA dysregulation in GI cells critical for normal gut motility.

#### 3.1.1. ICC-Specific miRNA Dysregulation

ICCs are major contributors for the rhythmic motor activities throughout the GI tract [18,74]. They generate and propagate slow waves and transduce neural inputs from enteric neurons to SMCs [74]. Functional defects due to excessive autophagy and/or apoptosis of ICCs have been reported in DBGIs [10,19,55]. miRNA-222 mediates autophagy and apoptosis of ICCs via regulation of the c-kit/SCF signaling pathway in a murine STC model [55]. Furthermore, the authors validated the c-kit/SCF pathway through in vitro experiments utilizing both a miR-222 mimic and inhibitor [55]. Another study showed that increased expression of miR-222 results in functional loss of ICCs in the colon tissue of a murine postoperative ileus (POI) model [75]. This model had reduced protein expression levels of the receptor tyrosine kinase (KIT) in ICCs, which was negatively correlated with miR-222 levels. Further, acupuncture attenuates miR-222 overexpression due to the rescue of KIT expression, indicating the role of miR-222 in modulating ICC functional restoration. One study demonstrated that miR-551b regulates intracellular Ca^2+^ concentration in ICCs, suggesting a possible therapeutic target for GI dysmotility [56]. A recent study reported reduction of miR-10b-5p in an ICC-specific manner leads to gastroparesis and STC [28]. Further, the authors rescued these gut dysmotility phenotypes as well as the functional loss of ICCs by gain-of-function strategies through intervention with a miR-10b-5p mimic.

#### 3.1.2. SMC-Specific miRNA Dysregulation

SMCs undergo cellular plasticity and functional activity changes in gut motility disorders [76]. miRNAs regulate both the cellular plasticity and functional activity of SMCs [23]. Additionally, they are critical for growth, differentiation, and survival of SMCs within the GI tract. Previous studies have evidenced that the inhibition of SMC-specific miRNAs triggers impairment in contractile properties resulting in smooth muscle thinning and intestinal dilatation [57,76,77]. miR-143 and miR-145 are involved in determination of the SMCs cellular fate and phenotype switching under different pathophysiological conditions [57]. Electrical excitability and electromechanical coupling are fundamental properties of SMCs for which ion channels are crucially important [78]. In SMCs, where Ca^2+^ signaling is critical for excitability, ion channels such as Na^+^ channels (NaV 1.5) contribute to the regulation of electrophysiological function [79]. miRNAs regulate ion channel densities in SMCs; however, inhibition of these ion channels leads to STC [25,26,80]. One study on STC showed that expression of the sodium voltage-gated channel alpha subunit 5 (SCN5A)-encoded NaV 1.5 was decreased in colonic tissues [23]. The authors found let-7f regulates NaV 1.5 channel expression in STC, which reduced the NaV 1.5 channel expression and NaV 1.5 currents in the SMCs from both humans and rats. These studies highlight the impact of miRNAs in the pathologies of SMCs that contribute to the development of DGBIs, such as STC.

#### 3.1.3. Enteric Neuron-Specific miRNA Dysregulation

Functioning of enteric neurons, such as nitrergic and cholinergic neurons, are critical for gut motor function [81,82]. Previous studies elucidated the roles of miRNAs in neuronal cell survival in pathological conditions of neurodegenerative diseases [83,84]. For instance, miR-433 and miR-9 play a role in the development of Parkinson’s disease and Huntington’s chorea, respectively [83].

Enteric neurons from diabetic mice fed with a high fat diet (HFD) have increased expression of miR-375, which directly targets 3-phosphoinositide-dependent protein kinase-1 (Pdk1) protein expression, a critical step in the activation of apoptosis [59]. The authors confirmed the detrimental effect of miR-375 on enteric neurons by transfecting cultured enteric neurons with a miR-375 inhibitor, which alleviated enteric neuronal apoptosis. In contrast, treatment with a miR-375 mimic induced enteric neuronal cell apoptosis and delayed gut transit. Furthermore, treatment with a miR-375 inhibitor to mice fed with a HFD for 5 weeks prevented the deleterious effects of the HFD on the number of enteric neurons as well as gut motility. These data provided direct evidence that miR-375 may be a possible therapeutic candidate for DGBIs.

#### 3.1.4. Gut Immune Cell-Specific miRNA Dysregulation

A significant contributing factor for developing DGBIs is gut immune dysfunction [85,86,87,88]. Further, gut motility is altered in patients with miRNA dysregulation in both mast cells and macrophages [50,60,62]. One study found that patients with STC had a negative correlation between miR-128 expression and the number of macrophages in colonic samples [62]. This may imply that increased macrophages in STC may be caused by decreased miR-128 expression. Another study used a high-throughput microarray to demonstrate the importance of miRNA expression in IBS-D [60]. The authors found patients with IBS-D had elevated expression levels of miR-490 and revealed the key role of this miRNA in IBS-D related immune dysfunction. Further, apoptosis and inhibition of proliferation of mast cells was caused by the inhibition of miR-490 expression, which acts through the proteinase-activated receptor 2 signaling pathway.

### 3.2. miRNA Dysregulation in Pathophysiological Mechanisms of DGBIs

#### 3.2.1. miRNA Dysregulation and Immune Dysfunction

Both adaptive and innate immune responses can be influenced by the regulation of immune cell differentiation via miRNAs [89]. Irregularities in these regulatory processes might cause a chronic inflammation condition, which is characteristic of the low-grade inflammatory mechanism of DGBIs [85,86,87,88]. Toll-like receptors (TLRs) and/or nucleotide-binding oligomerization domain-containing protein (NOD)-like receptors mediate innate immune responses, therefore, modulating downstream inflammatory cascades via pathways such as, nuclear factor kappa-light-chain-enhancer of activated B cells (NFκB), mitogen-activated protein (MAP) kinase, and other interferon regulatory factors [51]. Several studies have shown the importance of miRNAs in the regulation of these immune processes; for instance, miR-155 and miR-146a/b play a key role in TLR-signaling [90,91]. Further, miR-146a deficient mice had increased myeloid cell proliferation, developed autoimmune disorders, and tumorigenesis, while miR-155 deficient mice had functionally impaired dendritic cells [91]. Another study demonstrated suppression of let-7e along with upregulation miR-155 expression was caused by protein kinase B (Akt) deficient macrophages, which lead to the macrophages hyperresponsive reaction to lipopolysaccharides [92]. Further studies have demonstrated the interaction between miRNAs (miR-29, miR-192, miR-122, and miR-146a) and NOD2 in chronic GI inflammatory conditions [93]. One study showed overexpression of miR-155 regulates the differentiation of CD4^+^ T cells into Th1 cells [94]. In contrast, a miR-155 deficiency showed a tendency of CD4^+^ T cells to differentiate into Th2 cells. Further translational studies are warranted to clarify the function of miRNAs in gut immune regulation that contribute to the pathogenesis of DGBIs.

#### 3.2.2. miRNA Dysregulation and Visceral Hypersensitivity

The incidence and severity of DGBIs can be attributed to visceral hypersensitivity, which shows an increased perception of mechanical and/or chemical triggers applied to the gut [8,95]. This results in an amplification of the sensation of burning and pain in the GI. Transient receptor potential vanilloid 1 receptor (TRPV1), serotonin, transient receptor potential Ankyrin 1 channel (TRPA1), histamines, tachykinin, cannabinoids, protease-activated receptors, voltage-gated sodium and calcium channels, and acid-sensing ion channels are all modulators of visceral sensitivity [2,95]. The TRPV1 receptor is a peripheral nociceptor that is expressed on primary afferent sensory neurons [67]. A burning sensation and/or pain can be triggered by activation of these channels by capsaicin, acidic pH, thermal stimulus, and/or inflammatory mediators. Studies have shown a positive correlation between the upregulation of TRPV1 expression and abdominal pain in colonic biopsies from patients with IBS-D [67,96]. Further, miR-199a/b has been shown to regulate TRPV1 expression, which has a major role in visceral pain induction in patients with IBS-D [26]. This study evidenced that a reduction of miR-199a/b expression lead to upregulated TRPV1 expression, which therefore contributed to chronic visceral pain and nociception in patients with IBS-D. The authors showed that following stress and/or inflammation in the colon, TRPV1 enhanced visceral sensation and mechano-sensation in murine IBS models. Furthermore, a murine model of visceral hypersensitivity showed a reduction of miR-199 expression in the colon and dorsal root ganglion; however, this visceral nociception was reversed through gain-of-function administration of miR-199. Another mechanistic study demonstrated that upregulation of miR-495 inhibits the phosphoinositide-3-kinase (PI3K)/AKT pathway through targeting phosphatidylinositol 3-kinase 2 (PKIB), which reduces visceral sensitivity in a murine model of IBS-D [66]. Another study showed that miR-200a may induce visceral hyperalgesia by downregulating the cannabinoid receptor 1 (CNR1) and the serotonin transporter (SERT) leading to the development of IBS-D in mice [67].

#### 3.2.3. miRNA Dysregulation and Impaired GI Barrier Function

The mucosa-associated immune system is responsible for detecting and getting rid of toxic and immunogenic molecules at the gut luminal–mucosal interface [8]. When there is GI barrier dysfunction, there is activation of immune and inflammatory responses, which affect gut motility and visceral sensation in accordance to the degree of inflammatory response [97]. In order to maintain gut barrier integrity, proper expression of tight junction proteins is critical to keep enterocytes sealed and regulate intestinal permeability [51]. Recently, probe-based confocal laser endomicroscopy (pCLE), a novel in vivo method that allows cross-sectional imaging of the epithelium at a resolution of ~1 µm has allowed the assessment of epithelial gaps, in addition to assessing GI mucosal histology at both the cellular and subcellular levels [98]. Exploiting pCLE, duodenal mucosa of patients with IBS challenged with food antigens have increased intervillous spaces and epithelial breaks compared to healthy controls (HCs) [99]. Tight junction proteins, responsible for maintaining the integrity of the gut barrier, including zonula occludens-1 (ZO-1) and claudin-1 (CLDN1), are regulated via miRNAs. This regulation has been explored as a possible molecular mechanism of impaired GI barrier function [51]. Recently, it has been shown that miR-29a is involved in the pathophysiology of IBS-D, by downregulating the expression of ZO-1 and CLDN1 [68]. This study suggested that ZO-1 and CLDN1 expression in the colonic mucosa of an IBS-D murine model was significantly increased following intervention with a miR-29a inhibitor, suggesting miR-29a is critical to maintain intestinal barrier integrity. It also demonstrated that miR-29a is an important miRNA in regulating intestinal barrier integrity in patients with IBS-D through its interaction with the glutamine synthetase gene [69]. Upregulation of miR-29a leads to reduced glutamine synthetase (GLUL) levels resulting in impaired intestinal membrane permeability in patients with IBS-D [69]. Glutamine plays a crucial role in the maintenance of intestinal barrier function in animals and humans. Depletion of glutamine leads to villus atrophy and decreased expression of tight junction proteins resulting in increased intestinal permeability [100]. Ex vivo glutamine administration increased the expression of CLDN1 in the colonic mucosa of patients with IBS-D; however, further research is warranted to evaluate glutamine supplementation in clinical practice [100]. Another study revealed the important role of miR-122a in tumor necrosis factor-alpha (TNF-α) regulation and intestinal barrier integrity [70,101]. This study showed TNF-α treatment increased expression levels of miR-122a in Caco-2 monolayers and the miR-122a inhibitor protected tight junction permeability, suggesting that the upregulation of miR-122a is critical for impaired barrier function [70,101]. In another study, the exploration of miRNA-dependent modulation of tight junction-specific proteins was achieved by combining mRNA sequencing analysis and miRNA expression profiles in the jejunal tissue of patients with IBS-D [27]. The authors found miR-125b and miR-16 expression were reduced resulting in increased expression of cingulin and claudin-2. The expression levels of miR-125b, miR-16, cingulin, and claudin-2 was correlated with IBS symptoms and mast cell hyperplasia [27]. Another study showed intestinal hyperpermeability was enhanced by miR-144 up-regulation and attenuated by miR-144 downregulation in IBS-D rat colonic epithelial cells [71]. One study showed miR-21-5p expression in intestinal epithelial cells (IECs) regulated intestinal epithelial permeability via ARF4. ARF4 suppression in the IEC line increased the expression of tight junction proteins and decreased intestinal epithelial permeability [72]. Additionally HT-29 cells cultured with IBS-derived serum exosomes were found to have increased cell permeability and miR-148b expression, which led to downregulation of the regulator of G-protein signaling 2 (RGS2) expression [73]. These findings supported the crucial role of miRNAs in the development of impaired intestinal barrier function, which is a core pathophysiological mechanism in DGBIs [30]. Exploring the interplay between miRNAs and tight junction proteins would open a window for putative therapeutic candidates that might reinforce the gut barrier integrity, consequently preventing or ameliorating inflammatory reactions and potentially re-establishing gut homeostasis.

#### 3.2.4. miRNA Dysregulation and Serotonergic Signaling

The pathogenesis of DGBIs is heavily regulated via serotonergic signaling [102,103]. Enterochromaffin (EC) cells in our gut produce 90% to 95% of serotonin (5-hydroxytryptamine, (5-HT)) in our body [104]. Serotonin is important for various aspects of gut physiology, such as gut motility, secretion, absorption, visceral sensation, chemical and mechanical sensing, as well as neuronal signaling within the gut–brain axis [95,103,104,105]. These functions rely on the interaction of serotonin and 5-HT receptors, which are located on ICCs, SMCs, enteric neurons, enterocytes, and platelets [102,106]. It has been demonstrated that patients with IBS-D commonly have a single nucleotide polymorphism (rs201253747) in the 5-HT4 receptor (HTR4) gene [63]. This single nucleotide polymorphism blocks the binding site for miR-16 and miR-103/107 within the HTR4b/i isoforms, which may lead to inhibition of HTR4 expression. This study also found that symptoms of IBS-D were associated with the downregulation of miR-103 and miR-16 in the jejunum. An additional study found that female patients with IBS-D had a reduction in the c.76G>A variant of HTR3E, which is the miR-510 binding site [64]. This suggests the possible regulation of 5-HT3E subunit expression by miR-510. The authors also suggested that neural signaling, which causes many symptoms linked to IBS-D, may be associated with the upregulation of 5-HT3E. Moreover, a connection between miRNAs and SERT in patients with IBS and murine IBS models was revealed [65]. This study found patients with IBS had an increase in expression of miR-24 in IECs that was associated with the regulation of SERT expression, which has been proposed to play a role in the progression of visceral hypersensitivity.

### 3.3. miRNA Dysregulation and DGBIs

DGBIs are complex and heterogenous in nature and understanding their pathophysiological mechanisms remains elusive [2]. Moreover, though some treatment options are effective, these are inadequate for prolonged use due to poor tolerance and negative ramifications. There is an overwhelming demand to unearth pathophysiology-directed treatment regimens and diagnostic molecular markers for these conditions. Notably, miRNAs would be excellent biomarkers for DGBIs [51]. Further studies are warranted to illuminate miRNA-related biomarkers and the clinical application of miRNAs in screening for DGBIs and novel therapeutic options for DGBIs.

#### 3.3.1. miRNA Dysregulation in IBS

Recently, miRNA transcriptome data from colonic biopsies showed that miR-363-3p and miR-338-3p were downregulated, whereas miR-106b-5p and miR-532-5p were upregulated in IBS as compared to HCs [29]. miR-219-5p levels were decreased threefold in patients with IBS-D compared with HCs. The authors demonstrated inhibition of miR-219a-5p in IECs altered the expression of permeability-associated genes including TJP1/ZO-1, E-cadherin (CDH1), carcinoembryonic antigen-related cell adhesion molecule 5 (CEACAM5), and catenin delta 1 (CTNND1). In a miR-338-3p inhibition model of IECs, authors showed that the deregulated genes were significantly associated with GO terms including kinase and MAPK or protein serine/threonine kinase pathway genes [29]. MAPK pathway associated genes; MAPK1, MAPK8IP3, and MAPK9 were upregulated, and kinase inhibitor associated genes; tribbles related protein 3 (TRIB3), were downregulated. TRIB3 inhibits key inflammatory signaling pathways, including the MAPK and phosphatidylinositol 3 kinase networks: suggesting activation of the MAPK pathway. MAPK-associated genes, including stratifin (SFN, keratinocyte-releasable 14-3-3-sigma) and fatty acid amide hydrolase (FAAH), were shown to be upregulated [29]. SFN is a proinflammatory cytokine that binds to CD13 (also known as aminopeptidase N) and plays a role in pain sensation, which contributes to the development of IBS symptoms [107]. Another study showed elevated circulatory miR-342 and miR-150 in patients with IBS [108]. Additionally, miR-150 was found to be linked with GI inflammation and visceral pain. This may be due to the interaction between miR-150 and the AKT2-mediated inflammatory pathway. Further, miR-342 has been shown to target the genes involved in colonic motility and pain signaling [108]. One study showed miRNA profiling in microvesicles and revealed upregulation of miR-29a in patients with IBS, suggesting profiling of miRNAs in microvesicles might lead to a putative biomarker for patients with IBS [109]. Several studies suggested low-grade inflammation as a major contributing factor for IBS, particularly IBS-D [34,87]. Moreover, miRNA dysregulation in immune cells play an important role in inflammation [60,62]. Key miRNAs for instance miR-106a, miR-24, miR-26a, miR-21, miR-27a, miR-326, miR-155, miR-146a, miR-150, miR-221, and miR-181a regulate immune cell functions through release of pro-and/or anti-inflammatory cytokines, suggesting their possible role in IBS [51,61,89]. Nevertheless, the lack of reproducibility (most likely due to small sample sizes), the selection of varying regions in the gut for study, and different methodologies used to characterize miRNAs lead to conflicting results for the role of miRNAs in IBS.

#### 3.3.2. miRNA Dysregulation in FD

Exosomal miRNAs have been observed as possible biomarkers of FD [110]. One study found that miR-933 expression in gastric aspirate was down-regulated in patients with FD compared to HCs [110]. In addition, miR-933 expression levels negatively corelated with dyspeptic symptom scores and the frequency of epigastric burning and/or pain. Another study showed increased levels of miR-19a in a murine FD model, while reduced expression of miR-19 ameliorated the GI motility [111]. One study demonstrated that the polymorphism in miR-325 is associated with the susceptibility of FD [112]. This study also suggested pri-miR-325 polymorphisms lead to interactions with the solute carrier family 6 member 4 (SLC6A4) causing susceptibility of FD. Another study demonstrated that GI smooth muscle-specific miRNAs (miR-133 and miR-1) were significantly decreased in the stomachs of mice after chronic *Helicobacter pylori* infection, which led to delayed gastric emptying [113].

#### 3.3.3. miRNA Dysregulation in Gastroparesis

A recent study demonstrated that 6 out of 30 duodenal mucosal biopsies from patients with diabetic gastroparesis had differentially expressed miRNAs [114]. The miRNA target filter identified 248 anticorrelated miRNA-mRNA pairs. Thirty-six out of two hundred and forty-eight miRNA-mRNA pairs included 17 miRNAs that target mRNAs, which code for mitochondrial proteins. The miRNAs that were predicted to regulate differentially expressed OXPHOS genes included miR-101, miR-193b, miR-29c, miR-451a, miR-582, and miR-7974 [114]. Another study, elucidating the role of NFE2-related factor 2 (NRF2) and phase II antioxidant enzymes in gastric neuronal nitric oxide synthase (nNOS) function investigated whether supplementation of sepiapterin (SEP), a precursor for tetrahydrobiopterin (BH4) (a cofactor of NOS), restores altered nitrergic systems and redox balance in diabetic female rats [58]. The authors showed dietary SEP supplementation significantly reverted diabetes-induced changes in nNOS dimerization and function such as, nitric oxide (NO) downstream signaling molecules; HSP-90, a key regulator of nNOSα activity and dimerization; miRNA-28 that targets NRF2 mRNA, and levels of miRNA biogenesis pathway components, such as DGCR8 (Di-George Syndrome Critical Region Gene 8). This study identified a new pathway, wherein SEP regulates NRF2 mRNA turnover by suppressing elevated miRNA-28, which could be related to alterations in miRNA biogenesis [58]. One state-of-the-art study evidenced the fundamental role of miRNAs in the pathogenesis of diabetes and GI dysmotility and demonstrated deficiency of miR-10b-5p in pancreatic beta-cells and ICCs in mice concurrently triggered diabetes and gut dysmotility, respectively [28]. Most importantly, this pattern was also observed in patients with diabetic gastroparesis. Furthermore, this study suggested that a miR-10b-5p mimic could be clinically beneficial because it has more profound and prolonged effects in lowering blood glucose and improving GI motility in diabetic mice when compared to anti-diabetic and prokinetic medications. This study is clinically important because currently available treatments for DGBIs only relieve symptoms, which is simply not enough for clinical care of the patients [28]. These studies provided future perspectives to elucidate effective treatment regimens for gastroparesis, targeting the underlying pathophysiology rather than only symptomatic treatment.

#### 3.3.4. miRNA Dysregulation in STC

One study identified differentially expressed genes and miRNAs in colon tissue from patients with STC and found differential expression of 464 genes and 10 miRNAs [115]. Furthermore, to elucidate miRNA-mediated inflammatory signaling, authors found major histocompatibility complex, class II, DR Beta 5 (HLA-DRB5), C3, HLA-DRB1, and intercellular adhesion molecule (ICAM) were remarkably enriched, thus confirming inflammatory pathways in the pathogenesis of STC [115]. Another study explored the characterization of key miRNAs in STC using a bioinformatics platform of weighted gene correlation network analysis (WGCNA). WGCNA is an established tool to identify and depict modules comprising highly clustered genes and to correlate these genes modules with different diseases [50]. The authors found that miR-128 was significantly reduced in STC samples compared to controls. They showed correlation between the key miRNAs and STC (positive correlation: miR-619 and negative correlation: miR-20b, miR-486, miR-129, miR-30b, and miR-340) [50]. One recent study showed a small group of miRNAs is upregulated in STC, and many of these miRNAs target the SCN5A-encoded Na^+^ channel NaV1.5 [23]. Increased let-7f, a novel NaV1.5 regulator, expression led to decreased NaV1.5 expression and current density as well as reduced motility of GI smooth muscle [23]. These reports suggested miRNAs as novel diagnostic markers and potential therapeutic targets in STC.

## 4. miRNA-GPCR Interactions in DGBIs

Guanine nucleotide-binding proteins (G proteins) are essential for stimulus-response in ∼80% of all known membrane receptors that are linked to intracellular signal transduction pathways [116]. G protein-coupled receptors (GPCRs) are critical to normal gut functions, and their ligands include different types of molecules, such as neuropeptides, endocannabinoids, and chemokines [117,118]. Activation of GPCR signaling in the gut regulates many cellular and physiological processes, such as immune function, visceral sensation, absorption, secretion, and motility [116,118]. Moreover, GPCR signaling pathways are also targets of several gut function regulating medicines that work through receptors for opioids, acetylcholine, glutamate, serotonin, and dopamine [119,120]. Corticotropin-releasing hormone (CRH), through its receptors, CRF1 (localized in myenteric and submucosal nervous plexus of the distal gut) and CRF2 (localized in the luminal surface of crypts and myenteric neurons), is an important mediator in stress-related physiological responses from the GPCR class B family [121,122]. Besides CRH, other ligands of the CRF receptors include urocortin (UCN), UCN2, and UCN3 [120].

Acute stress induces glucocorticoid feedback and reduces CRF1 receptor expression by increasing miR-449a levels in the rat pituitary [123]. In addition, UCN2 increases miR-325 expression in rat pituitary cells in vitro and suppresses luteinizing hormone in the rat pituitary during acute stress [124]. Opioid receptors belong to the GPCR class A family and are drug targets to relieve symptoms in patients with DGBIs [119,125]. Opioid receptors are expressed in the central and enteric nervous systems (ENS) via OPRM1-, OPRK1-, and OPRD1-receptors [119]. Along the entire gut, opioid-receptor signaling activation regulates visceral sensation, motility, and secretion [119,126]. Recent studies on opioid tolerance in human neuroblastoma cells in vitro and in mouse models showed morphine treatment in vitro and in vivo increases expression of let-7 and miR-103/-107, which reduces opioid-receptor expression by directly targeting sequences on the 3′-UTR of the opioid receptor [127,128]. miR-134 regulates opioid-receptor expression in human neuroblastoma cells in vitro, whereas miR-134 expression is reduced in the dorsal root ganglia in a murine chronic inflammatory pain model, resulting in overexpression of opioid-receptor [129]. Taken together, GPCR signaling activation in the central nervous system (CNS) and ENS are associated with dysregulated miRNA expression in acute and chronic stress conditions mediating alternations in gut functions. Further research on GPCR-miRNA interactions in the ENS will enhance our understanding of the pathogenesis and therapeutic options for DGBIs.

## 5. Clinical Insight and Therapeutic Alternatives for DGBIs

Gut microbial alterations are extensively prevalent in patients with DGBIs [95]. Some studies showed evidence of the role of host miRNAs in shaping the gut microbiota, which might further modulate the altered gut microbiota-linked pathophysiological mechanisms such as gut motility, visceral hypersensitivity, gut immune dysfunction, impaired gut barrier function, and altered gut–brain axis [72,130,131,132,133,134]. The commensal bacteria in the gut are essential in health and disease. However, little is known about how they are naturally regulated or strategies to manipulate them. A landmark study showed that host fecal miRNA directly regulates gut microbial gene expression and growth [130]. This study showed that human and mouse feces contained specific miRNAs, such as miR-155 and miR-1224. Mice deficient in the miRNA-generating protein Dicer (restricted only to intestinal epithelial cells) had reduced levels of fecal miRNA, suggesting that IECs are a significant source of miRNA in feces. Further, the authors showed that mice with Dicer knocked out specifically in IECs had gut microbiota dysbiosis and were more susceptible to induced colitis than wild-type mice. This study provided evidence that the host can actively modulate gut microbes through miRNAs [130]. Another study showed that fecal miRNAs might be used as indicators of imbalance at the host–microbe interface [131]. The authors found that the expression of let-7b, miR-141, and miR-200a in feces from germ-free (GF) animals was lower than conventional mice, suggesting miRNAs can be used as an independent, non-invasive marker of microbial fluctuations along with gut pathology in the intestine [131]. Further, one study compared the miRNA profile of IECs from conventional and GF mice and demonstrated that miR-21-5p is induced by commensal bacteria, with implications for intestinal barrier function regulation [72]. Studies are scant on host miRNA–gut microbiota crosstalk in DGBIs. Future studies are warranted to provide significant clinical insights into different facets of the host miRNA–gut microbiota interplay and to elucidate the role of the host miRNAs in shaping gut microbial composition influencing physiological and pathophysiological mechanisms in DGBIs.

### 5.1. Gut Microbiota and DGBIs

Food digestion, metabolite synthesis, pathogenic microorganism colonization prevention, essential vitamin production, drug metabolism, removal of toxic substances, regulation of the gut–immune response, and maintenance of gut homeostasis all rely on the proper functioning of the gut microbiota [135,136]. An imbalanced gut microbiota (dysbiosis) can lead to many physiological and metabolic changes that contribute to the development of many human disorders [137]. DGBIs, including IBS and FD, have been shown to correlate with gut microbial dysbiosis, which is characterized by a shift in the proportion of commensal to pathogenic microorganisms, an increase in the amount of small intestinal bacteria, as well as growth of colonic bacteria in the small intestine [95,138]. Additionally, the progression of many disease states is influenced by numerous factors such as, the host immune response, host physiology, host diet, host environment, microbial produced metabolites, and microbe–microbe interactions [139].

Microbe–host interactions (i.e., immune and metabolic responses) have become further understood in recent years and have brought to light the importance of these interactions on pathological factors associated with DGBIs [31,95]. Gut microbial dysbiosis activates a mucosal immune response that leads to gut epithelial barrier dysfunction, which gives rise to visceral hypersensitivity and gut dysmotility (hallmarks of DGBIs) [31,95]. Patients with IBS have been shown to have considerable alterations to their gut microbiota when compared to HCs [140,141]. However, future studies are necessary to confirm if the relationship between DBGIs and the alteration of the gut microbiota is an association or causation. Previous studies have demonstrated that a microbiome “signature” consisting of reduced microbial diversity as well as an increase in the abundance of *Clostridium* spp. and methanogenic bacteria is prevalent in patients with IBS [142,143]. Gut motility has been shown to be accelerated by *Clostridium* spp. due to their ability to increse synthesis of serotonin [143]. Additionally, increased abundance of *Methanobravibacter smithii* has been found in fecal samples from patients with functional constipation and constipation-pre-dominant IBS (IBS-C) [144]. Further, it has been demonstrated that patients with IBS have an increased ratio of *Firmicutes* to *Bacteroidetes* and *Streptococcus* and *Ruminococcus*, as well as a decreased ratio of *Lactobacillus* and *Bifidobacterium* species [145]. Duodenal mucosal samples from FD patients have a significant reduction of *Veillonella*, *Prevotella*, and *Actinomyces* when compared to HCs [146]. Moreover, gastric fluid from FD patients showed an absence of *Acidobacteria* and an increased ratio of *Bacteroidetes* to *Proteobacteria* [147]. Taken together, altered gut microbial signaling has been at the forefront of understanding the microbiota-related pathogenesis of DGBIs.

### 5.2. Gut Microbiota-Derived Molecules and DGBIs

Gut microbiota-derived metabolites constantly send signals to host organs regulating physiological properties of health and disease [16,139,148]. The major fermentation products produced by the gut microbiota are short-chain fatty acids (SCFAs) that provide approximately 10% of energy required by the host. Butyrate, propionate, and acetate are the most commonly studied SCFAs produced by the gut microbiota and have been shown to bind to G-protein-coupled receptors (GPR43 and GPR41), also known as free fatty acid receptor 2 and 3 (FFAR2 and FFAR3), respectively [149,150]. Induced expression of the hormone peptide YY through the binding of SCFAs to FFAR3 has been shown to improve impaired gut motility [150]. This is of great importance because it allows the host to harvest more of the available energy from food. A recent study has highlighted the importance of SCFAs in GI motility by demonstrating that patients with IBS-C had decreased levels of the SCFAs butyrate and propionate and patients with IBS-D had increased levels of butyrate when compared to HCs [151]. Another study demonstrated that SCFAs produced by the gut microbiota in both humans and mice stimulate EC cells by increasing tryptophan hydroxylase 1 (Tph1) expression and therefore serotonin production in the gut [152,153]. Tryptamine is a monoamine metabolite, similar to serotonin, that is produced from tryptophan by the hosts gut bacteria (i.e., *Clostridium sporogenes* and *Ruminococcus gnavus*) and is present in large quantities in both human and rodent feces [154]. Further, a recent study demonstrated that the 5-HT4 receptors, located in the epithelium of the colon, are responsible for facilitating the role of tryptamine in the GI tract [155]. The authors showed that an engineered strain of *Bacteroides thetaiotaomicron* that produces tryptamine, improved GI motility in mice [155]. Taken together, microbe-derived metabolites are able to impact many aspects of host physiology and therefore, could be used for more effective, preventative, and therapeutic interventions for DGBIs.

### 5.3. Gut Microbiota Regulated Modulation of the Gut–Brain Axis in DGBIs

Interactions between the gut and the brain are bi-directional [156]. The brain has an essential role in maintaining proper gut function and the gut has been shown to greatly influence brain function [156]. Bi-directional gut–brain interactions serve as important modulators of GI functionality influencing motility, gastric secretions, immune activity, visceral sensations, and intestinal barrier functions [157]. Gut-to-brain bi-directional signaling can also modulate the gut functions through indirect signaling between the gut microbiota and the host [157]. Impaired epithelial barrier function, caused by the gut microbiota-related immune response, results in altered gut–brain interactions [158,159]. The perception of gut stimuli and modulation of various gut functions are conducted through the emotional motor system [157]. Gut microbial alterations therefore, could modulate the function of neurotransmitters, such as dopamine, serotonin, γ-aminobutyric acid, and acetylcholine, either by synthesis or consumption, resulting in changes in emotional state and behavior [157,160].

Psychological conditions, including stress, anxiety, and depression, are associated with DGBIs and contribute to the pathophysiology as part of an integrated biopsychosocial model [161]. This model highlights the significance of the bidirectional communication of the gut–brain axis in regard to DGBIs [161]. Functional brain MRI studies in patients with DGBIs demonstrate abnormalities of structural and functional networks in areas of the brain responsible for processing information such as the vagovagal reflux and visceral motor system [9,12]. The brain alters gut physiology, such as visceral sensitivity and motility, mediating symptoms of DGBIs [156]. Meanwhile, changes in the gut provide feedback to the brain, influencing psychological health [156]. Interestingly, there is a high prevalence of both depressive and anxiety (23%), anxiety (39%), and depressive (29%) disorders in IBS patients [162]. Further, anxiety is associated with impaired gastric accommodation and correlates negatively with gastric discomfort and pain thresholds, as well as gastric compliance in patients with hypersensitive FD [12]. Taken together, psychological comorbidity could be a consequence of a chronic GI disease burden and reduced quality of life, and its role in the pathophysiology of DGBIs is still unequivocally underevaluated.

### 5.4. Gut Microbiome Modulation through Probiotic Intervention and Personized Nutrition

Probiotics, live microorganisms, are believed to have favorable effects on the gut microbiota. However, the few clinical studies to date elucidating the efficacy of probiotic use lack consistency. Specific strains of probiotics, such as *Bifidobacterium lactis* DN-173, *Bifidobacterium animalis* DN-173010154, and multispecies probiotics have shown favorable effects in patients with IBS [163]. Further, IBS-C patients showed accelerated gut transit and improved symptoms after they were treated with probiotics containing *Bifidobacterium lactis* [163]. Moreover, FD patients have been shown to have improved upper gut symptoms after treatment with probiotic *Lactobacillus* strain [164]. One study reported nanoceria combined with probiotic strains *Lactobacillus casei* IMV B-7280 and *Bifidobacterium animalis* VKB, and *Bifidobacterium animals* VKL normalized the gut microbiota of mice fed with a fat-enriched diet [165]. Further, prebiotic (inulin-type fructans) intervention in mice with high dietary protein levels led to increased abundance of of *Bifidobacteria* and reduction of *Desulfovibrio* spp., which prevented the production of proteolytic metabolites typically seen in these mice [166]. Probiotics have also demonstrated substantial effects in personalized medicine. For example, lactic acid producing bacteria (*Lactobacillus acidophilus* IMV B-7279, *L. casei* IMV B-7280, *L. delbrueckii* subsp. *bulgaricus* IMV B-7281, *L. rhamnosus* LB-3 VK6, *L. delbrueckii* LE VK8, *L. plantarum* LM VK7) and *Bifidobacteria* strains (adhesive ability, resistance to antibiotics, and gut biological fluids) have been shown to be effective in modulating the microbiota according to the patient’s phenotype as well as their individual needs [167]. Taken together, the studies presented demonstrate the potential for probiotic treatments for DGBIs, specifically, disorders with pathophysiological mechanisms that are related to gut microbial dysbiosis.

The hosts diet greatly impacts the abundance of specific microorganisms as it is the main source of fuel for our microbiota [168]. Changes in the host’s diet will also alter the gut microbial composition, which therefore leads to flucuations in the host’s physiology [169]. Therefore, dietary intervention should not be overlooked as a powerful tool to help patients overcome microbial dysbiosis [170]. Patients suffering from conditions as a result of gut dysbiosis have abdominal pain and bloating due to the translocation of microbes able to ferment carbohydrates and produce excess gas from the colon to the small bowel [171,172]. However, switching to a diet low in fermentable oligosaccharides, disaccharides, monosaccharides, and polyols (FODMAP), has been shown to improve symptoms of IBS and of dysbiosis [171,173]. Additionally, reduced total abundance of bacteria has been found in patients that consumed low FODMAP diets [173,174]. Further, growth of less pathogenic microorganisms was found in patients that consumed a diet high in complex carbohydrates when compared to patients who had a diet rich in protein and/or fat [168]. Moreover, diets rich in fiber have been shown to increase SCFAs production and inhibit the colonization of potentially pathogenic bacteria [172,175]. Further studies are warranted to more accurately elucidate the effects of different diets on the composition of the gut microbiota.

Modern approaches utilizing a rigorous, robust design, large and multicentric longitudinal human studies might be exploited to integrate multiomic data, including gut physiological and pathophysiological mechanisms for personalized treatment options. Such insight would lead to better stratification of therapeutic approaches, leading to the targeted restoration of altered gut–brain functions in patients with DGBIs.

## 6. miRNAs as a Potential Therapeutic Option

The identification of dysregulated miRNAs and enhancement in the understanding of the pathways regulated by miRNAs suggest the prospect of their use as therapeutic alternatives. The intervention through miRNA mimics, which may be useful when a particular miRNA is deficient or absent or the inhibition of abnormally upregulated miRNAs through miRNA inhibitors are promising therapeutic approaches (Figure 1). One advantage of miRNA-mediated therapeutics is that it is simply reintroducing the endogenous miRNA, which may be a safer treatment compared to traditional synthesized chemicals. Moreover, miRNAs can modulate the physiological and pathophysiological cellular functions in the gut (Figure 1C) (Table 1). Their regulatory role on the expression of multiple proteins may place these molecules in a prime position for their utility in the prognosis, diagnosis, and treatment of DGBIs. Increased understanding of miRNA dysregulation at the cellular level will shed a brighter light on the molecular mechanisms in the pathogenesis of these disorders. Although miRNA research has advanced with a plethora of data elucidating miRNAs in the pathogenesis of DGBIs, more robust miRNA-based translational studies are warranted to explore the pathogenesis and therapeutics more thoroughly.

## 7. Current Challenges and Solutions for Mirna Therapeutics

Over the past decade, few miRNA-based therapies have proceeded to clinical trials of the several miRNA-based therapies that have been developed; however, there are a few pharmaceutical companies trying miRNA-based therapies for diabetes, hepatocellular carcinoma, hepatitis C virus infection, and nonalcoholic fatty liver disease. Many of these studies are only in phase 1 or 2 of clinical trials or are still in the pre-clinical phase [176]. miRNAs as therapeutics for DGBIs are still in their infancy; nevertheless, there is hope for future clinical trials promoting the promising use of an anti-sense oligonucleotide-based strategy centered around miRNAs as a therapeutic approach.

Current challenges for miRNA therapeutics include: (i) Cell specificity; it is almost impossible to find a miRNA that is expressed solely in one particular cell type and is important for disease development. The quality of an RNA therapeutic is determined by the strength of its on-target specificity as well as the absence of off-target and undesired on-target effects. On one hand, miRNAs are known to target multiple different mRNAs [177]. On the other hand, each mRNA target is regulated by multiple miRNAs and may possess different miRNA binding sites in close vicinity, resulting in binding competition [178]. It should be noted that miRNAs are not gene-specific and also target other yet to be explored biological pathways. It would be of great interest to explore if the dysregulation of miRNAs in the IECs is also reflected in cell-derived extra-cellular vesicles, such as exosomes [179]. Exosomes, 40–100 nm nanosized vesicles that are released from almost all cell types into blood or urine, have been shown to act as mediators for cell-to-cell communication because of the regulatory functions of their content [180]. Moreover, exosomes are found abundantly in several body fluids making them a potential source of non-invasive biomarkers for pathophysiological mechanisms underlying DGBIs. One possible approach to limit the detrimental overexpression of a miRNA in undesirable cells, is to express the RNA therapeutic using a suitable vector under the control of a specific promoter, which is overexpressed only in the cells of interest [38]. (ii) The hurdles of delivery; the delivery vehicles for miRNAs confer long-term stability of the therapeutic candidate and enable cell/tissue-specific delivery; therefore, minimizing possible off-target adverse effects. Efficient delivery of miRNA therapeutics, not only to the organ and cell type of interest, but also across the cell membrane to perform their intracellular functions is one of the greatest challenges in the field. Accordingly, the first and foremost reason for clinical trial termination regarding miRNA therapeutics is lack of efficacy. Efficient delivery of miRNAs is challenging owing to their instability, negative charge and hydrophilic nature preventing diffusion through cell membranes [181]. First- and second-generation chemical modifications improve stability and uptake by inducing resistance to nuclease degradation and increasing interaction with proteins [182]. Third-generation antisense technologies such as neutrally charged phosphorodiamidate morpholino oligomers (PMOs) are highly stable but face difficulties with cellular uptake and require administration in high doses [183]. Different delivery systems are also being used, including lipid- and polymer-based vectors and ligand–oligonucleotide conjugate delivery systems [184]. These delivery systems are taken up via different endocytosis mechanisms and consequently, endosomal escape of the miRNA therapeutic must be facilitated to prevent its lysosomal degradation [184]. Further challenges include the lack of reproducibility when comparing miRNA profiling data obtained using different platforms and even intra-platform variation is common [185,186]. For example, absolute miRNA expression levels have substantial variation between various platforms; however, the relative quantification of miRNAs tend to be very similar between platforms. The variation in absolute miRNA expression is typically due to low abundance of miRNAs in the sample along with different sensitivities of different platforms. To combat this problem, only the top 50 most highly expressed miRNAs may be analyzed to obtain less cross-platform variation; however, different platforms may not select the same 50 miRNAs and may bias certain miRNAs [186]. Further, the advantages of RNA sequencing over other miRNA quantification methods may not outweigh the numerous biases that make quantification less reliable (e.g., adapter ligation bias, reverse transcription bias, and amplification bias) [187,188]. It is recommended to use unique molecular identifiers (UMIs) to diminish these biases and improve the efficacy and reliability of miRNA quantification [187].

## 8. Future Directions

The unprecedented interdisciplinary approach and collaborative effort between researchers in the field of DGBIs and bioinformaticians is required for thrilling miRNA discoveries in the future. miRNA therapeutic candidates must be extensively tested for immunogenicity, chemically modified to improve pharmacokinetics and pharmacodynamics, delivered under consideration of biodistribution patterns and intracellular escape mechanisms, specifically interact with the intended target, and be dosed at an optimal level to trigger the desired effect. Integrating the information of databases (miRBase, TargetScan, Ingenuity Pathway Analysis,) would help researchers to accurately predict miRNAs and their target genes relevant to pathophysiological conditions [189,190]. Databases for the pathogenesis of DGBIs are necessary for future research in the field of DGBIs.

Therapeutic candidates for DGBIs may be unearthed through robust elucidation of the regulatory mechanisms of miRNAs and their target genes on the modulation of gut functions, such as gut motility, GI barrier function, visceral sensation, and gut immune function. However, the causes of miRNA dysregulation in DGBIs remain elusive. The speculations for the cause of miRNA dysregulation might include alterations in diet, the gut microbiota and its derived molecules, and epigenetic modulators due to changes in the cellular microenvironment in DGBIs [59]. Diet plays a crucial role in altering the expression of miRNAs in the gut, along with additional physiological consequences [59,191,192]. It is also possible that the microbiome either dependently or independently of diet may alter the miRNA expression patterns in patients with DGBIs [59,191,193,194,195]. The mechanism may involve microbial-derived metabolites such as short-chain fatty acids and bile acids that modulate gut motility and indirectly alter miRNA expression [196]. Although challenging, with the many new, promising, and creative developments that are emerging through valuable preclinical work, the challenges faced in the field of miRNA therapeutics will ultimately be overcome. There is considerable promise for the use of RNA-based medicines, such as RNA interference-based therapeutics and RNA-based vaccines, for the improved functioning of GI cells. Future interdisciplinary development of these therapeutics to improve tolerance, specificity, and cell/tissue specific delivery of these medicines will provide substantial hope for the clinical care of patients with DGBIs.

## Figures and Tables

**Table 1:** Dysregulated miRNAs in DGBIs.

Disease	miRNA (Expression)	Target			Key Findings	References
		Gene	Cell	Pathophysiological Mechanisms		
Gastroparesis, STC	miR-10b ↓	KLF11, KIT	ICCs	GI dysmotility	Deficiency of miR-10b in ICCs led to gastroparesis and STC, while injection of miR-10b rescued the dysmotility.	[28]
STC	miR-222 ↑	KIT, SCF	ICCs	GI dysmotility	Overexpression of miR-222 in ICCs diminished ICC proliferation and accelerated autophagy, whereas inhibition of miR-222 prevented apoptosis of ICCs.	[55]
Delayed gut transit	miR-551b ↓	KIT	ICCs	GI dysmotility	miR-551b mimic increased intracellular Ca^2+^ concentration in ICCs.	[56]
STC	let-7f ↑	NaV1.5	SMCs	GI dysmotility	Upregulation of let-7f resulted in decreased NaV1.5 expression, current density, and reduced motility of GI smooth muscle.	[23]
Delayed gut transit	miR-143/145 ↓	SRF	SMCs	GI dysmotility	Deficiency of Dicer in SMCs resulted in degeneration of SMCs in GI smooth muscle. SRF-induced miR-143 and miR-145 expression promoted GI SMC differentiation and suppression of proliferation.	[57]
Delayed gut transit	miR-28 ↑	NRF2	Enteric neurons	GI dysmotility	Elevated miR-28 levels in enteric neurons delayed gastric motility by modulating nNOSα dimerization.	[58]
Delayed gut transit	miR-375 ↑	Pdk1	Enteric neurons	GI dysmotility	Overexpression of miR-375 in enteric neurons resulted in neuronal cell apoptosis while injection of a miR-375 inhibitor prevented the neuronal cell apoptosis and improved gut motility.	[59]
IBS-D	miR-490 ↑	Tryptase, PAR-2	Mast cells	Gut immune dysfunction	Overexpression of miR-490 in mast cells resulted in increased proliferation of mast cells while inhibition of miR-490 expression promoted apoptosis and inhibited proliferation of mast cells.	[60]
IBS model	miR-181c ↓	IL-1A	Colonic biopsy	Gut immune dysfunction	Inhibition of miR-181c resulted in increased IL-1A levels while overexpressed miR-18c silenced IL-1A and inhibited low-grade inflammation in IBS rats.	[61]
STC	miR-128 ↑	MAPK-14	Colonic macrophages	Gut immune dysfunction	miR-128 expression negatively correlated with macrophage number, suggesting a miR-128 inhibitor might be a potential therapeutic candidate for a subset of patients with STC having gut immune dysfunction.	[62]
IBS-D	miR-16, miR-103/107 ↓	5HTR4	Jejunal biopsy	Altered serotonin signaling	miR-16 and miR-103/107 were downregulated in jejunum biopsies from IBS-D patients and were negatively correlated with IBS symptoms.	[63]
IBS-D	miR-510 ↑	5HTR3E	IECs	Altered serotonin signaling	miR-510 expression was upregulated in enterocytes and myenteric plexuses of colon sections from patients with IBS-D and resulted in altered serotonin signaling via 5HTR3.	[64]
IBS	miR-24 ↑	SERT	IECs	Altered serotonin signaling/visceral hypersensitivity	miR-24 expression was upregulated in colonic biopsies from IBS patients. Treatment with a miR-24 inhibitor increased nociceptive threshold levels and reduced MPO activity in the proximal colon of IBS mice, and upregulated expression levels of SERT in IECs.	[65]
IBS-D	miR-199a/b ↓	TRPV1	Colonic biopsy	Visceral hypersensitivity	Decreased colonic miR-199a/b correlated with visceral pain in patients with IBS-D. Administration of miR-199 (lenti-miR-199 precursor) reversed visceral nociception in a rat model of visceral hypersensitivity.	[26]
IBS-D	miR-495 ↓	PI3K, AKT, PKB	Rectal biopsy	Visceral hypersensitivity	miR-495 upregulation reduced visceral sensitivity in IBS-D mice via inhibition of the PI3K/AKT signaling pathway by targeting PKIB.	[66]
IBS-D	miR-200a ↑	CNR1, SERT	Colonic biopsy	Visceral hypersensitivity	Upregulation of miR-200a induced visceral hyperalgesia by targeting CNR1 and SERT. A miR-200a mimic markedly inhibited the expression of CNR1/SERT in IBS-D rats.	[67]
IBS	miR-338 ↓	MAPK, threonine kinase	IECs	Visceral hypersensitivity	Inhibition of miR-338 increased MAPK or protein serine/threonine kinase pathway genes leading to increased visceral sensation.	[29]
IBS-D	miR-29a ↑	ZO-1, CLDN1	Colonic biopsy	Intestinal barrier dysfunction	Upregulation of miR-29a downregulates ZO-1 and CLDN1 expression resulting in leaky gut. Treatment with miRNA-29a inhibitor downregulated D-LA and DAO activity, and increased the expression of ZO-1 and CLDN1 in the intestinal mucosal epithelium.	[68]
IBS-D	miR-29a ↑	GLUL	Colonic and duodenal biopsy	Intestinal barrier dysfunction	Upregulation of miR-29a led to reduced GLUL levels resulting in impaired intestinal membrane permeability in patients with IBS-D.	[69]
IBS	miR-219a ↓	TJP1/ZO-1, E-CDH1, CEACAM5, CTNND1	IECs	Intestinal barrier dysfunction	Inhibition of miR-219a-5p in intestinal epithelial cells led to hyperpermeability as TEER was reduced and dextran flux was increased.	[29]
IBS model	miR-122a ↑	TNF-α	IECs	Intestinal barrier dysfunction	miR-122a upregulation led to intestinal barrier dysfunction via TNF-α-mediated degradation of occludin.	[70]
IBS model	miR-144 ↑	OCLN, ZO-1	Colonic biopsy	Intestinal barrier dysfunction	miR-144 upregulation led to intestinal hyperpermeability, while inhibition of miR-144 improved intestinal barrier function in IBS-D rat colonic epithelial cells.	[71]
IBS model	miR-21 ↓	PTEN, PDCD4, ARF4	IECs	Intestinal barrier dysfunction	Inhibition of miR-21-5p in IECs led to intestinal epithelial hyperpermeability.	[72]
IBS-D	miR-125b, ↑ miR-16	CGN, CLDN2	IECs	Intestinal barrier dysfunction	Upregulation of miR-125b and miR-16 downregulated CGN and CLDN2 and resulted in intestinal barrier dysfunction.	[27]
IBS	miR-148b ↑	RGS2	HT-29 cells cultured with IBS-derived serum exosomes	Intestinal barrier dysfunction	miR-148b overexpression increased cell permeability and downregulated RGS2 expression.	[73]

Abbreviations: AKT/PKB, protein kinase B; ARF4, ADP-ribosylation factor-4; CDH1, cadherin-1; CEACAM5, carcinoembryonic antigen-related cell adhesion molecule-5; CGN, cingulin; CLDN1, claudin-1; CNR1, cannabinoid receptor-1; CTNND1, catenin delta-1; GI, gastrointestinal; GLUL, glutamine synthetase; HFD, high fat diet; IBS-D, diarrhea-predominant irritable bowel syndrome; ICCs, interstitial cells of Cajal; IECs, intestinal epithelial cells; IL-1, interleukin-1; KIT, receptor tyrosine kinase; KLF11, krüppel-like factor-11; nNOS, neuronal nitric oxide synthase; MAPK, mitogen-activated protein kinase; miRNA, microRNA; NaV1.5, voltage-gated sodium channel subunit 1.5; NRF2, nuclear factor erythroid 2-related factor; OCLN, occludin; PAR-2, protease activated receptor-2; PDCD4, programmed cell death-4; PDK1, 3-phosphoinositide-dependent protein kinase-1; PI3K phosphoinositide-3-kinase; PTEN, phosphatase and tensin homolog; RGS2, regulator of G-protein signaling-2; SCF, stem cell factor; SERT serotonin transporter; SMCs smooth muscle cells; SRF, serum response factor; STC, slow transit constipation; TEER, trans-epithelial electrical resistance; TJP, tight junction protein; TNF-α, tumor necrosis factor-alpha; TRPV1, transient receptor potential vanilloid-1 receptor; ZO-1, zonula occludens-1; 5-HTR3E, 5-hydroxytryptamine receptor 3 isoform E.

## Data Availability

Not applicable.
